# Novel synthesis approach for highly crystalline CrCl_3_/MoS_2_ van der Waals heterostructures unaffected by strain†

**DOI:** 10.1039/d4na00935e

**Published:** 2025-02-21

**Authors:** Mahmoud M. Hammo, Samuel Froeschke, Golam Haider, Daniel Wolf, Alexey Popov, Bernd Büchner, Michael Mertig, Silke Hampel

**Affiliations:** a Leibniz Institute for Solid State and Materials Research Dresden Helmholtzstraße 20 01069 Dresden Germany m.hammo@ifw-dresden.de +49 17687315637; b Institute of Physical Chemistry, Technische Universität Dresden 01062 Dresden Germany; c Institute of Solid State and Materials Physics, Technische Universität Dresden Dresden Germany; d Kurt-Schwabe-Institut für Mess- und Sensortechnik Meinsberg e.V. Kurt-Schwabe-Straße 4 Waldheim 04736 Germany

## Abstract

Controlling the layer-by-layer chemistry and structure of nanomaterials remains a crucial focus in nanoscience and nanoengineering. Specifically, the integration of atomically thin semiconductors with antiferromagnetic two-dimensional materials holds great promise for advancing research. In this work, we successfully demonstrate a new synthesis approach for high-crystallinity CrCl_3_/MoS_2_ van der Waals heterostructures *via* a thermodynamically optimized chemical vapor transport (CVT) process on *c*-sapphire (0001) substrates. The 2H-MoS_2_ layers can be grown as monolayers or with varying twist angles whereas the deposition of CrCl_3_ layers in a second step forms the well-defined heterostructure. Of particular significance are the sharp and clean edges and faces of the crystals, indicating high-quality interfaces in the heterostructures. Raman spectroscopy, AFM and HRTEM confirm the monocrystalline character and precise structure of these layered nanomaterials, in which their intrinsic properties are preserved and unaffected by strain. This can pave the way for next-generation applications, particularly in valleytronics, opto-spintronics, and quantum information processing.

## Abstract 2

Synthesis of high crystalline CrCl_3_/MoS_2_ van der Waals heterostructures *via* chemical vapor transport approach.

## Graphical abstract

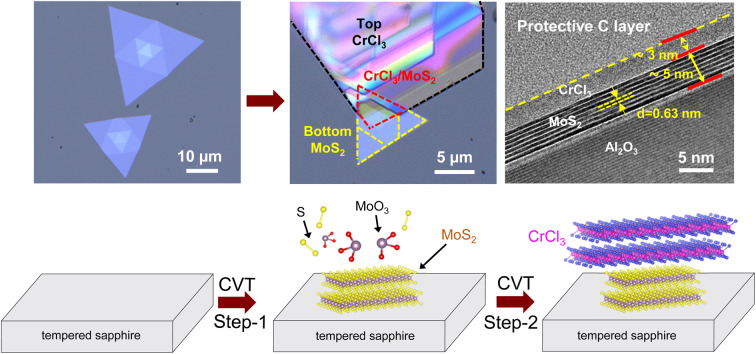

## Introduction

The exceptional properties of van der Waals (vdW) heterostructures, which comprise atomically thin layered materials such as graphene, transition metal trihalides (TMTHs), transition metal dichalcogenides (TMDCs) and various topologically layered materials, have paved the way for a wide range of innovative research opportunities, encompassing both fundamental and applied research.^1–6^ These heterostructures are predominantly created by stacking two-dimensional (2D) materials layer-by-layer. The weak vdW forces enable the assembly of dissimilar materials without the constraints of lattice matching.^7^ A number of studies have demonstrated that the incorporation of a monolayer (ML) of TMDCs into 2D materials, including WSe_2_/CrBr_3_, can lead to a notable alteration in their optoelectronic properties.^8–12^ However, the synthesis of strain-free heterostructures remains an intriguing area of study. By employing a synthesis method that mitigates strain, we ensure that the intrinsic properties of layers are preserved, resulting in high-quality interfaces with minimal defects. The strain-free synthesis method is driven by its significance for practical applications, particularly in valleytronics, opto-spintronics, and quantum information processing. In valleytronic devices, the ability to manipulate electron populations in distinct energy valleys of 2D semiconductors such as MoS_2_ is crucial. Strain-induced distortions can shift these valleys, reducing device efficiency and stability.^13^ The strain-free growth ensures that the valleys remain well-defined, enabling robust valley-dependent charge transport and optical transitions. In addition to the well-studied heterostructure WSe_2_/CrBr_3_, the construction and investigation of the similar heterostructure CrCl_3_/MoS_2_ is also very promising, for example, to explore phenomena such as the spin proximity effect. The combination of a 2D semiconductor (MoS_2_) and a layered A-type antiferromagnetic insulator (CrCl_3_)^14–16^ forms a promising heterostructure for opto-spintronics. The direct bandgap of MoS_2_ supports efficient light emission and absorption, while CrCl_3_ provides magnetic ordering. This enables optical control of spin and valley degrees of freedom, paving the way for spin-based photonic devices and memory elements.^17^ Therefore, the combination of these types of materials continues to generate significant interest among physicists, chemists and material scientists,^18–21^ and represents an effective approach to developing highly functional materials for spintronic and opto-spintronic applications.^19,22^ High-quality, strain-free interfaces are essential for fabricating devices that leverage quantum coherence. In heterostructures where spin–orbit coupling or exciton recombination is involved, strain can introduce unwanted decoherence or scatter carriers.^23^

Two principal approaches are typically applied for the fabrication of vdW heterostructures. The first one is a top-down approach, which encompasses techniques such as exfoliation from the bulk/single crystal and subsequent assembly through the utilization of standard dry transfer techniques.^18–20,24–27^ This approach does not allow for precise control over the number of layers, which limits its overall significance. In addition to being time-consuming, this approach presents other disadvantages, including damage to the edge structure and contamination of the cleaved interfaces. Furthermore, the presence of process-related impurities at the interface, including polymer residues,^28^ water and air bubbles,^29^ impedes the efficient formation of heterostructures. This significantly affects the overall quality of the heterostructure.^30^

The second approach is a bottom-up method, in which such heterostructures are commonly grown using techniques such as chemical vapor deposition (CVD),^31,32^ physical vapor deposition (PVD),^33^ or various epitaxial processes. These methods are particularly well-suited for the production of heterostructures with high-quality, clean, and atomically sharp interfaces. While the synthesis *via* CVD or PVD requires rather complex equipment, the synthesis *via* CVD is a highly cost-effective and straightforward process. Instead of these methods, and for the first time, sequential chemical vapor transport (CVT) was employed to prepare 2D vdW heterostructures. Sequential CVT comprises two consecutive steps. Each step is designed to achieve a specific part of the total vdW heterostructure growth. The optimal parameters (*e.g.* precursor deposition, temperature gradient Δ*T*, residence time and transport time) have to be identified for each individual step.

This study describes a scalable two-step vapor phase growth process for the fabrication of highly crystalline, vertically stacked CrCl_3_/MoS_2_ heterostructures on *c*-sapphire (Al_2_O_3_) substrates oriented along the (0001) direction. Different growth temperatures and times have been evaluated, and the best conditions have been identified. Theoretical studies of gas phase composition and equilibria support the experimental performance. The resulting heterostructures were analyzed by optical microscopy, atomic force microscopy (AFM), Raman spectroscopy and high-resolution transmission electron microscopy (HRTEM). Of particular significance are the sharp and clean edges and faces of the crystals, indicating high-quality interfaces of the heterostructures.

## Experimental section

### Materials

CrCl_3_ (Alfa Aesar, anhydrous 99.9%), MoO_3_ (99.9995%, thermo scientific, melting point: 795 °C), S (metals basis 99.9995%, thermo scientific, boiling point: ∼445 °C), and KCl (99.999%, Sigma Aldrich).

### Thermodynamic simulations using TRAGMIN software

Thermodynamic simulations were performed with a modified version of the software “TRAGMIN 5.1”. The used thermodynamic data of all species were taken from the FactPS database^34^ and a list of the used species is given in the ESI.† A system volume of 9.4 mL was used, and 5 × 10^−9^ mmol H_2_O and 1 × 10^−9^ mmol Ar traces were added for all simulations.

### Preparation and pretreatment of substrates

The (0001) plane of *c*-sapphire wafers were cut into substrates with specific dimensions (10.0 × 5 × 0.5 mm^3^). A photoresist was spun onto the wafers to protect the polished surface from damage during the cutting process. To remove the photoresist afterwards, the substrates were rinsed with acetone and cleaned by ultrasonic treatment in distilled water for 15 min. Afterwards, they were rinsed again with distilled water and excess liquid was removed with compressed nitrogen. Finally, the substrates were annealed in air as described in Fig. S1.†

### Synthesis of CrCl_3_/MoS_2_ by sequential CVT

For the synthesis of MoS_2_ nanolayers, the starting materials were mixed in a glovebox with the ratio MoO_3_ : S = 1 : 2. One milligram of this mixture was placed on the source side of the ampoule, with approximately 1 milligram of KCl utilized as the transport agent. A *c*-sapphire (0001) substrate was positioned on the sink side of the ampoule and sealed using a vacuum sealing line (oxyhydrogen flame) at a pressure of ∼2 × 10^−3^ mbar. For the growth process, a two-zone LOBA furnace (HTM Reetz, GmbH) was set to the following temperatures *T*_1_ = 1000 °C; *T*_2_ = 800 °C for 30 min, followed by natural cooling. The ampoules were opened in the glovebox, and the substrate was transferred to a new ampoule. In the second ampoule, approximately 5 milligrams of CrCl_3_ were placed on the source side and the substrate was transferred to the sink side. The growth conditions for the second step were 600 °C at the source side and 500 °C at the sink side as shown in Fig. S2.† The ampoule was quenched in water after 15 min.

### Preparation of the TEM lamella

To analyze the cross-section of the CrCl_3_/MoS_2_ heterostructure using TEM, a lamella in the overlapped region of the CrCl_3_/MoS_2_ heterostructure was prepared by focused ion beam (FIB) cutting. The preparation was carried out using a Helios 5 CX (Thermo Scientific). Initially, the sample was coated with a 20 nm carbon layer using a sputter coater, followed by electron beam-induced deposition (EBID) and ion beam-induced deposition (IBID) for additional protection and stability. The FIB cutting was performed at an acceleration voltage of 30 kV and a current of 2.5 nA to create the initial trenches, followed by a lower current for the final polishing to ensure minimal damage to the sample. This multi-step coating and cutting process ensured the sample's integrity during FIB milling and subsequent TEM analysis. The precise FIB technique allowed for the accurate extraction of a thin lamella, which is crucial for detailed cross-sectional TEM studies.

### Characterization

#### Optical microscopy

After breaking the ampoule, the first characterization of the crystal morphology was conducted using an optical microscope (Keyence VHX-7000) equipped with a VHX-7020 CMOS image sensor.

#### Scanning electron microscopy (SEM) and energy dispersive X-ray spectroscopy (EDX)

Morphological and compositional analyses were performed using scanning electron microscopy (SEM) at various magnifications, coupled with energy dispersive X-ray spectroscopy (EDX) using an FEI Nova-NanoSEM 200. The EDAX Genesis Spectrum software was used to measure the composition of the crystals. 5 measurements at different spots/crystals were averaged to calculate the composition of each crystal or experiment.

#### Raman spectroscopic investigations

These measurements were performed using a T64000 Raman Spectrometer (Horiba Jobin Yvon) under 532 nm laser excitation. The spectra were recorded at room temperature utilizing an 1800 g mm^−1^ grating.

#### Transmission electron microscopy (TEM)

As-grown MoS_2_ layers were transferred onto TEM grids (Lacey-carbon 200 mesh Cu, Plano GmbH). Briefly, MoS_2_ on the *c*-sapphire (0001) substrate was immersed in 500 μL of pure ethanol and subjected to ultrasonic treatment for 10 min. Subsequently, several drops of the resulting solution were pipetted onto the TEM grid and allowed to air dry. Then the transferred crystals were examined under an optical microscope before TEM investigation. For the CrCl_3_/MoS_2_ heterostructure, the lamella was prepared by FIB cutting and then measured by TEM. The measurements were performed with a HRTEM “FEI Titan^3^ 80-300” (ThermoFisher Scientific Company), operated at an acceleration voltage of 300 kV, with selected area electron diffraction (SAED) conducted over an area of a few nm.

#### Atomic force microscopy (AFM)

Atomic force microscopy analysis was conducted in tapping mode under ambient conditions using TESPA-V2 tips on a “Dimension ICON” with ScanAsyst (Bruker, USA). Data analysis was conducted using the “Nanoscope Analysis” software, version 1.8.

## Theoretical basis

**Fig. 1:**
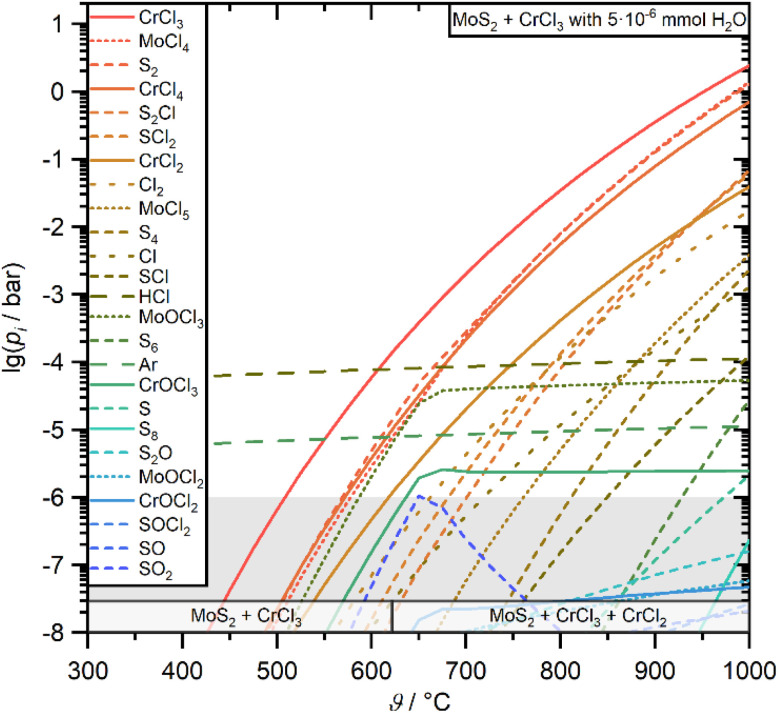
Calculation results of the temperature-dependent vapor pressures of the dominant vapor species over an equimolar mixture of MoS_2_ and CrCl_3_ with traces of water. The predicted condensed phases at the bottom are displayed for amounts larger than 10^−5^ mmol. The grey area indicates vapor pressures that are too low to contribute to transport processes.

To investigate the thermodynamic stability of two potential compounds in one system that are so different in their chemistry such as MoS_2_ and CrCl_3_, thermodynamic equilibrium calculations of the complex chemical systems with inclusion of the vapor phase have been performed. The initial results for the temperature-dependent vapor pressures and condensed phase stabilities of an equimolar CrCl_3_/MoS_2_ system are displayed in Fig. 1.

The calculation results demonstrate that, in principle, CrCl_3_ and MoS_2_ are stable next to each other over a wide temperature range suitable for the vapor phase growth of the desired heterostructures. However above *ca.* 625 °C a significant decomposition of CrCl_3_ to CrCl_2_ sets in but has no major influence on the predicted vapor pressures.

**Fig. 2:**
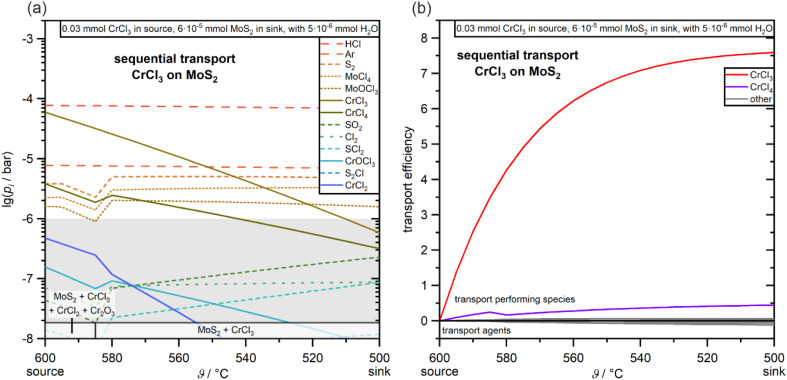
(a) Temperature-dependent partial pressures and predicted deposited phases for the simulation of the sequential CVT heterostructure deposition of CrCl_3_ (starting material in the source) on MoS_2_ (starting material in the sink). For this simulation, the source temperature was fixed at 600 °C, while the temperature of the sink was varied between 595 and 500 °C. The grey area indicates vapor pressures that are too low to contribute to transport processes. (b) Calculated transport efficiencies for this simulated CVT.

Based on this initial assessment of the thermodynamic stability of the heterostructure, more detailed investigations of potential deposition strategies were performed. While studies on the individual deposition of ultrathin nanosheets of CrCl_3_ by CVT have already been reported,^35–37^ and can be adapted for the deposition of the first underlying layer of the heterostructure in a sequential deposition strategy, the optimization of the second deposition step on top of the first layer presents an even greater challenge due to the potentially much more complex chemical equilibria in the combined system. To understand this second potential deposition step and to estimate a suitable parameter window for following practical experiments, further calculations of thermodynamic equilibria were combined with CVT models to simulate the transport processes. The primary results for the investigated sequential deposition of CrCl_3_ on MoS_2_ are displayed in Fig. 2a. Despite the very small amount of MoS_2_ used in this simulation to mimic a potentially thin layer of MoS_2_ on the substrate, the simulations confirm that a regular deposition of CrCl_3_ on MoS_2_ should be possible at *T*_source_ above *ca.* 550 °C, similar to the individual deposition of CrCl_3_ nanosheets.^35^ The calculated transport efficiencies (Fig. 2b) further confirm that the transport process of CrCl_3_ is dominated by sublimation and that, despite the relatively high partial pressures of Mo-containing vapor species (Fig. 2a), the transport process is not strongly interfered with.

The sequential transport in the opposite order (MoS_2_ on CrCl_3_) with the use of KCl as a transport agent could not be simulated properly because the use of KCl most likely generates vapor-phase complexes that are relevant for the transport process but are unknown with respect to both their exact structure and thermodynamic data.^38,39^

## Results and discussion

### Growth of MoS_2_ as the bottom layer of the heterostructure

To optimize the growth of few-layer MoS_2_ crystals as a bottom layer *via* CVT, the precursors molybdenum trioxide (MoO_3_), sulfur (S) and potassium chloride (KCl) were used. We varied the synthesis temperatures (500 °C to 1000 °C), the temperature gradient (50–200 °C) and the transport time (5–60 min).

**Fig. 3:**
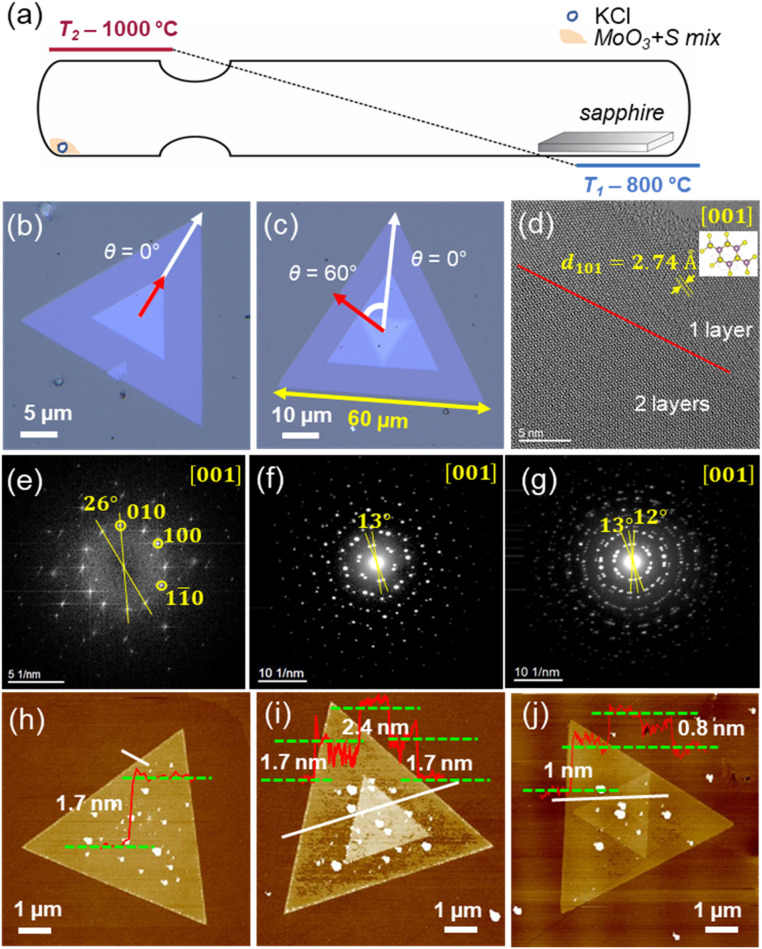
(a) Schematic CVT setup for growth of MoS_2_ nanostructures (heating rate 10 K min^−1^, *T*_2_ = 1000 °C, *T*_1_ = 800 °C, *t* = 30 min, naturally cooling). (b and c) Optical microscopy images of twisted MoS_2_ stacked in different sizes on a *c*-sapphire (0001) substrate. (d) HRTEM image recorded at the edge of a similar MoS_2_ flake at the 001 zone axis. The inset shows the unit cell in this orientation, where Mo is purple and S is yellow. The red line highlights the edge of a second layer twisted by 26° with respect to the first one. (e) Fourier transform of (d) with indexed reflection and the indicated twist angle between the two layers. (f and g) Electron diffraction patterns oriented along the 001 zone axis (hexagonal space group 194) of the same nanoflake at areas where two (f) and three (g) twisted layers are present. AFM images and height profile measurements of different MoS_2_ crystals along the marked white line showing three different shapes: triangular (h), parallel (0°) (i), and anti-parallel (60°) (j).

The optimal synthesis parameters include a heating rate of 10 K min^−1^, with the temperature range maintained between 800 °C and 1000 °C for a duration of 30 min, followed by natural cooling (Fig. 3a). This specific heating rate ensures a controlled and uniform temperature increase, which is crucial for achieving the desired crystalline quality and thickness. Maintaining the synthesis temperature within this range for 30 min allows adequate material deposition and layer formation, promoting the growth of few-layer MoS_2_ with minimal defects. The natural cooling process helps in stabilizing the crystal structure, thereby preserving the integrity and uniformity of the MoS_2_ layers.

### Characterization of MoS_2_

The MoS_2_ crystals are uniformly distributed across the substrate in various sizes and exhibit different shapes, including triangular, parallel (where triangular nanosheets stack at 0°), and anti-parallel (where triangular nanosheets stack at 60°). They are stable under ambient conditions. The lateral dimensions of the crystals vary from 10 to 60 μm. Optical images of selected crystals are shown in Fig. 3b and c. They exhibit sharp edges and show random twists at different angles: 0° (Fig. 3b) and 60° (Fig. 3c). The pale blue color resembles MoS_2_ with few layers while the color gradually saturates with an increasing number of layers. Notably, under the same temperature gradient conditions, but using a longer transport time (more than one hour), the crystals will become thicker. Additionally, SEM images further support these observations, as shown in Fig. S3.†

TEM measurements were conducted to confirm the crystallinity and crystal structure. The bright-field TEM image in Fig. S5b† displays a MoS_2_ flake, a few tens of nanometers thick, on the lacey carbon TEM grid. The mono-crystalline layer exhibits an almost hexagonal arrangement in this zone axis orientation (above the highlighted red line in Fig. 3d), whereas the superposition of two layers twisted by an in-plane rotation angle of 26° results in a prominent appearance of a so-called Moiré pattern (below the red line in Fig. 3d). In fact, the latter is confirmed by image simulations (see Fig. S6†) using the DrProbe software package,^40^ incorporating the twist angle, which is determined from the Fourier transform (Fig. 3e) of the entire flake from which the HRTEM image in Fig. 3d is shown. The diffraction patterns (Fig. 3f,g) recorded at the 001 zone axis orientation exhibit individual reflections that confirm the high crystallinity of the samples. Specifically, the two lines in Fig. 3f cross two reflection pairs, indicating a twisted double layer of MoS_2_ with a 13° twist angle. Similarly, three lines in Fig. 3g cross three reflection pairs, indicating a twisted multi-layer of MoS_2_ with twist angles of 12° and 13°. HRTEM images of a similar flake, also taken at the 001 zone axis orientation (hexagonal space group 194) reveal a twisting between two MoS_2_ layers, one extending until the upper edge and one ending 10 nanometers away from the edge. These observations, along with the measured *d*-spacing, confirm the high crystallinity and precise interlayer alignment of the MoS_2_ flakes, validating the twisted bilayer and multilayer structures. More TEM images and their Fourier transforms are provided in Fig. S5.†

The crystal thicknesses were determined by AFM measurements (Fig. 3h–j). Triangular shapes with a thickness of 1.7 nm are observed, corresponding to three layers (Fig. 3h). Parallel-oriented triangular nanosheets formed on top of the triangular MoS_2_ crystal, are also found to exhibit thicknesses of 1.7 nm, corresponding to three layers, or 2.4 nm, corresponding to four layers (Fig. 3i). Anti-parallel oriented nanosheets are found to have a thickness of 0.8 nm, corresponding to a MoS_2_ monolayer (Fig. 3j). The AFM images also provide a detailed topographical view of the nanosheets, on the one hand clearly showing the step heights corresponding to the different thicknesses. On the other hand, these high-resolution images highlight the uniformity and smoothness of the nanosheets.

### Growth of CrCl_3_/MoS_2_ heterostructures

**Fig. 4:**
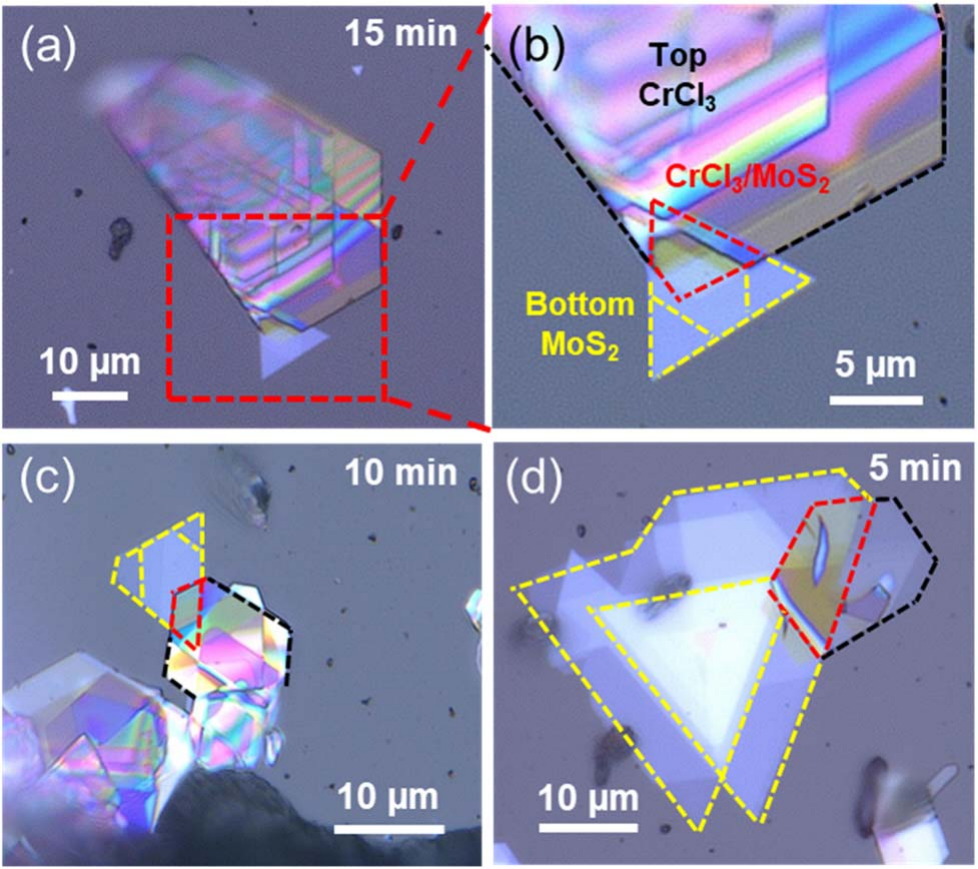
Optical microscope images of CrCl_3_/MoS_2_ heterostructures prepared by sequential CVT. (a) CrCl_3_/MoS_2_ heterostructure with an ∼5 nm CrCl_3_ layer in the overlapped region (CrCl_3_ was quenched after 15 min). (b) Zoomed-in view of the area marked in (a). (c) CrCl_3_/MoS_2_ heterostructure (CrCl_3_ was quenched after 10 min). (d) CrCl_3_/MoS_2_ heterostructure (CrCl_3_ was quenched after 5 min), illustrating the uniformity and alignment of the layers stacked atop each other (MoS_2_ trigonal shapes with yellow dashed lines and CrCl_3_ hexagonal shapes with black dashed lines).

In a second step, CrCl_3_/MoS_2_ heterostructures were prepared and analyzed. The sequential CVT approach involved the deposition of CrCl_3_ (as a top layer) in a vertical orientation on MoS_2_, which had already been grown on a *c*-sapphire (0001) substrate (see Fig. 4 and S8†). Here, purified CrCl_3_ (purification process described in Table S1†) was used. The growth conditions can be described as follows: *T*_1_ = 500 °C; *T*_2_ = 600 °C and quenched after 5, 10 or 15 min in tap water using an ampoule catcher.^35^ The CrCl_3_ crystals have a hexagonal shape with very sharp edges and exhibit colors ranging from blue to violet depending on their thickness. The CrCl_3_ crystals were precisely aligned with the MoS_2_ layer so that heterostructures were realized. EDX measurements confirming the composition of CrCl_3_ are provided in Fig. S7.† The thickness of CrCl_3_ crystals depends on the quenching time. After 5 min of quenching, CrCl_3_ crystals with a thickness of approximately 15 nm were obtained. If the ampoule is quenched after 2 hours or more, the CrCl_3_ crystals grow to over 200 nm in thickness. Thus, by varying the CVT conditions, the thickness of the CrCl_3_/MoS_2_ heterostructure can be controlled.

### Characterization of the CrCl_3_/MoS_2_ heterostructure

**Fig. 5:**
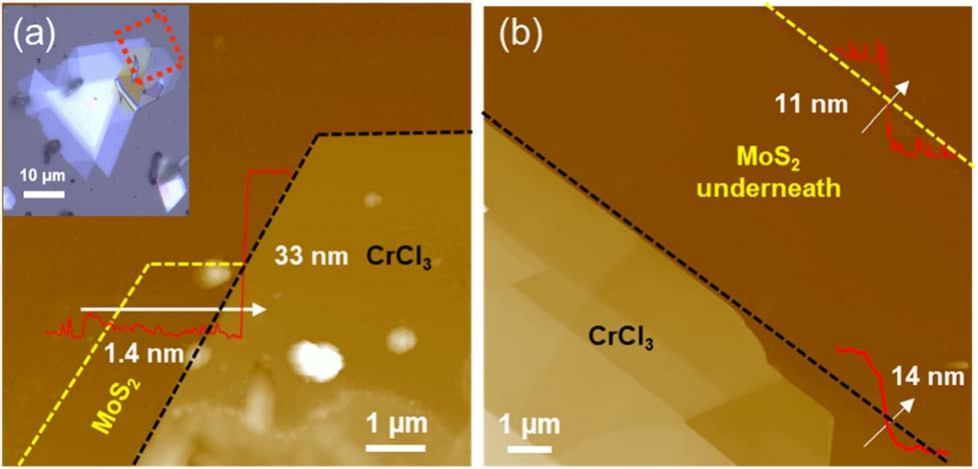
(a) AFM images of the CrCl_3_/MoS_2_ heterostructure for the marked region in the optical microscope image (inset in (a)). (b) AFM image of another CrCl_3_/MoS_2_ heterostructure. Inset: height profile along the white arrow, shown with the same *x*-scale as the image scale.

AFM images of a selected CrCl_3_/MoS_2_ heterostructure were taken to determine their thickness. The thickness of the MoS_2_ layer was determined to be 1.4 nm, corresponding to a bilayer, and the CrCl_3_ layer thickness was found to be 33 nm, corresponding to a multilayer stack, as shown in Fig. 5a. In another sample, shown in Fig. 5b, the thicknesses of the MoS_2_ and CrCl_3_ layers were found to be 11 nm and 14 nm, respectively. Clear step edges in the height profile, observed when crossing from the MoS_2_ layer to the CrCl_3_ layer, indicate that CrCl_3_ typically forms a flat stack on MoS_2_.

**Fig. 6:**
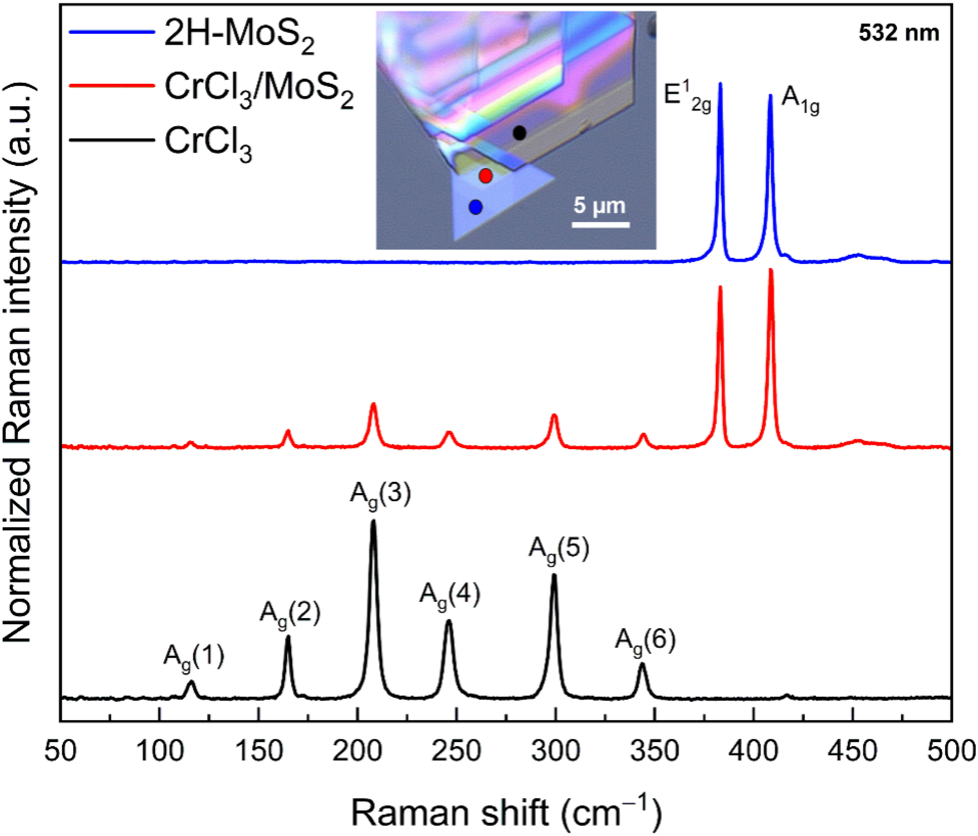
Raman spectra of the CrCl_3_/MoS_2_ vertical heterostructure. Raman spectra recorded from the three different regions, labelled in the inset optical microscope image of CrCl_3_/MoS_2_, showing the anti-parallel triangles of MoS_2_, and CrCl_3_ on the top. There are overlapping Raman signatures with MoS_2_ (red line).

Raman measurements were conducted on the as-grown MoS_2_ and CrCl_3_ layers, as well as the CrCl_3_/MoS_2_ heterostructure (as illustrated in Fig. 6 and Fig. S9†). The spectra were obtained from different regions, including MoS_2_, CrCl_3_, and the CrCl_3_/MoS_2_ heterostructure. The top blue spectrum, representing only MoS_2_, exhibits two prominent peaks corresponding to the E^1^_2g_ and A_1g_ vibration modes of 2H-MoS_2_. These peak positions are consistent with previous reports,^41,42^ and corroborate our obtained TEM results. The black spectrum at the bottom displays six modes that align with the monoclinic phase of the synthesized CrCl_3_ crystals.^16^ The middle red spectrum, recorded from the CrCl_3_/MoS_2_ heterostructure region, shows all characteristic peaks of both CrCl_3_ and MoS_2_.

Interestingly, no shift in the Raman modes was observed in the heterostructure region compared to the individual layers. Typically, the formation of a heterostructure results in interlayer charge transfer and strain to achieve potential equilibrium and compensate for lattice mismatch at the interface, which often modifies the properties of the individual layers.^12,19^ However, the absence of a shift in the E^1^_2g_ and A^1^_g_ modes suggests that the underlying MoS_2_ layer remains unaffected by strain and charge doping during the growth process. Similarly, the lack of apparent shift in the Raman modes of CrCl_3_ indicates a minimal amount of strain in CrCl_3_ due to lattice mismatch. So, this new synthesis approach can pave the way for innovative advances in growing nanostructures.

This stability in the Raman signal suggests that the semiconductor properties of MoS_2_ remain intact even after CrCl_3_ deposition. The preservation of the characteristic Raman peaks and their intensities indicates that the heterostructure retains the high crystalline quality and integrity of both the MoS_2_ and CrCl_3_ layers. This result confirms that the sequential CVT technique allows for the successful assembly of CrCl_3_/MoS_2_ heterostructures without affecting the crystallinity of MoS_2_.

**Fig. 7:**
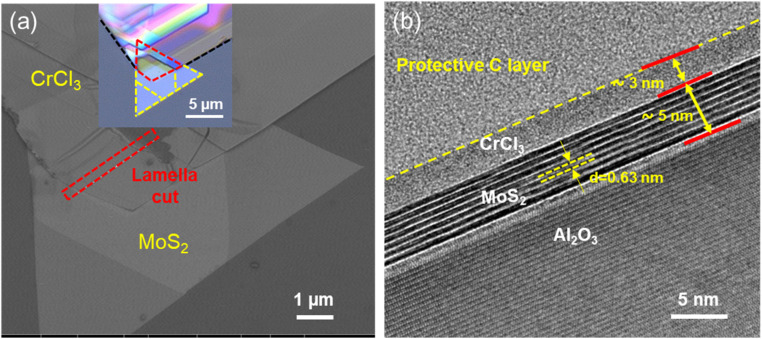
Structural characterization of the CrCl_3_/MoS_2_ heterostructure. (a) SEM image of the CrCl_3_/MoS_2_ heterostructure, highlighting the marked region for lamella cutting, with the corresponding optical microscope image (inset in (a)). (b) Cross-sectional HRTEM image of a vertically-staked CrCl_3_/MoS_2_ heterostructure. The image shows the distinct layers of MoS_2_ and CrCl_3_, highlighting their interface and alignment.

The SEM and cross-sectional HRTEM images of the CrCl_3_/MoS_2_ heterostructure (Fig. S10† and 7) depict how the different layers are attached to each other and the Al_2_O_3_ substrate. In Fig. 7, the 5 nm thin atomically flat 2H-MoS_2_ layer is most clearly visible, showing no indications of lattice impurities and point defects. The separation between different vdW layers remained intact and the *d*_003_-spacing between them was determined to be 0.63 nm. On top, an ∼3 nm thick layer of CrCl_3_ is identified, reflecting successful heterostructure formation. The hardness of the insulating Al_2_O_3_ substrate required the use of a higher voltage, *i.e.*, ion energy during FIB cutting. This process unfortunately amorphized the CrCl_3_ layer whereas MoS_2_ was more stable during this process. Additionally, CrCl_3_ presents a significant challenge due to its susceptibility to damage when exposed to high-energy electron beams and intense laser excitation.^43^

## Conclusions

In this study, the synthesis and characterization of CrCl_3_/MoS_2_ vdW heterostructures were comprehensively investigated on *c*-sapphire substrates using a thermodynamically optimized sequential CVT process. The realized structures of MoS_2_ exhibit heights down to about 0.8 nm and several μm in lateral size, as measured by AFM. Notably, some of these crystals are randomly twisted by different angles in the range of ∼0° to ∼60°. The majority of CrCl_3_ nanosheets exhibit a thickness ranging from 3 to 35 nm, as determined statistically by AFM for several CrCl_3_ crystals, with large lateral dimensions distributed across the substrate. Notably, thicker crystals are predominantly observed at the substrate edges, with some also present near the center. High-crystallinity and structural quality of both layers were confirmed through Raman spectroscopy and HRTEM. Interestingly, no shift in the Raman modes was observed in the heterostructure, which would typically appear due to strain and interlayer charge transfer to compensate for the lattice mismatch at the interface. These kinds of heterostructures create an optimistic outlook in studying some of the interesting physical properties, *e.g.*, magnetic proximity effects in TMDCs. Our method preserves structural integrity, promoting long spin and exciton lifetimes for use in quantum computing and secure communication technologies.

## Data availability

The data supporting this article have been included as part of the ESI.†

## Author contributions

Mahmoud M. Hammo: conceptualization, investigation, data curation, visualization, and original draft – writing – review & editing. Samuel Froeschke: simulation, – review & editing. Golam Haider: scientific discussion, and helped in modifying the manuscript in the publication form. Daniel Wolf: TEM measurements and analysis. Alexey Popov: Raman measurements. Bernd Büchner: supervision and proof reading. Michael Mertig: resources, acquisition, supervision and proof reading. Silke Hampel: conceptualization, resources, acquisition, and supervision. All authors have given approval to the final version of the manuscript.

## Conflicts of interest

The authors declare that there are no competing interests.

## Supplementary Material

NA-OLF-D4NA00935E-s001
