# Supplementary material for: Novel synthesis approach for highly crystalline CrCl3/MoS2 van der Waals heterostructures unaffected by strain
Source: Nanoscale Adv. 2025 Feb 21;7(8):2351–9. doi: 10.1039/d4na00935e (PMC11880837; doi:10.1039/d4na00935e)
Supplement: NA-007-D4NA00935E-s001 [file NA-007-D4NA00935E-s001.pdf]

Supplementary Information (SI) for Nanoscale Advances.
This journal is © The Royal Society of Chemistry 2025

Electronic Supplementary Information

Novel Synthesis Approach of Highly Crystalline $CrCl_3/MoS_2$ van der Waals Heterostructures Unaffected by Strain

Mahmoud M. Hammo *[ab], Samuel Froeschke [a], Golam Haider [a], Daniel Wolf [a], Alexey Popov [a], Bernd Büchner [a,c], Michael Mertig [b,d], Silke Hampel [a]

[a] Leibniz Institute for Solid State and Materials Research Dresden, Helmholtzstraße 20, 01069 Dresden, Germany

[b] Institute of Physical Chemistry, Technische Universität Dresden, 01062 Dresden, Germany

[c] Institute of Solid State and Materials Physics, Technische Universität Dresden, Dresden, Germany

[d] Kurt-Schwabe-Institut für Mess- und Sensortechnik Meinsberg e.V., Kurt-Schwabe-Straße 4, Waldheim 04736, Germany

Corresponding Author *E-mail: m.hammo@ifw-dresden.de

Purification of commercial $CrCl_3$

Firstly, we purified the commercial $CrCl_3$ from water and oxygen by CVT [1]. In brief, one−chamber ampoule was filled out separately with 1.0 g of commercial $CrCl_3$ and sealed under pressers ~ 2×10^{-3} mbar. Then, the ampoule was placed into two−zone furnace in recipe (see Table S1). We used powder grinder to grind $CrCl_3$ inside the glove box and used it directly for growing heterostructures.

	**Temperature gradient (°C)**	**Time (h)**
**Cleaning transport (Inverse temperature gradient)**	600 (source)−900 (sink)	5
**Actual transport**	800 (source)−600 (sink)	100
**Total time**		105

Table S1. Recipe for purification of commercial $CrCl_3$ by CVT in a muffle furnace.

Pre−treatment of substrates

c-sapphire (Al_2O_3) substrates oriented along (0001) direction

The synthesis of 2D materials is also significantly influenced by the substrate selection. Restrictions on lattice matching that are necessary for the epitaxial growth of conventional thin films are relaxed by weak vdW interactions between the 2D material and substrate. As a result, SiO_2 (300nm)/Si wafers are often preferred substrates due to their readily available back gate. However, for growth on wafer scale, crystalline substrates may offer greater appeal [2]. Therefore, we selected *c*-sapphire (0001) substrate (Crystal, GmbH) as a substrate in this work.

First of all, the photoresist film on the substrates was removed by washing it with acetone in small vessels and putting it in an ultrasonic bath for one minute. Then, rinsed with distilled water in the same way. Finally, we used the nitrogen spray gun to remove any drops of solution on our substrates. Before CVT and after cleaning processes, the substrates were annealed in air in a chamber furnace (Carbolite Gero) at temperature 1000 °C for 1 h, reaching a ultra-smooth and atomically flat surfaces (Figure S1) [3]. We used plastic tweezers for all handling to avoid scratching the substrate.

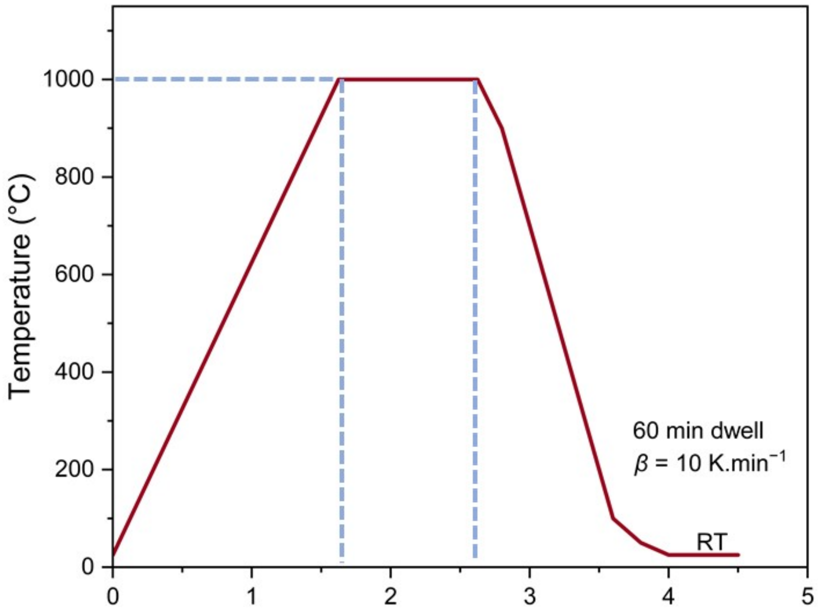

Figure S1. Temperature curve during annealing of *c*-sapphire (0001) in the chamber furnace at 1000 °C for 1h.

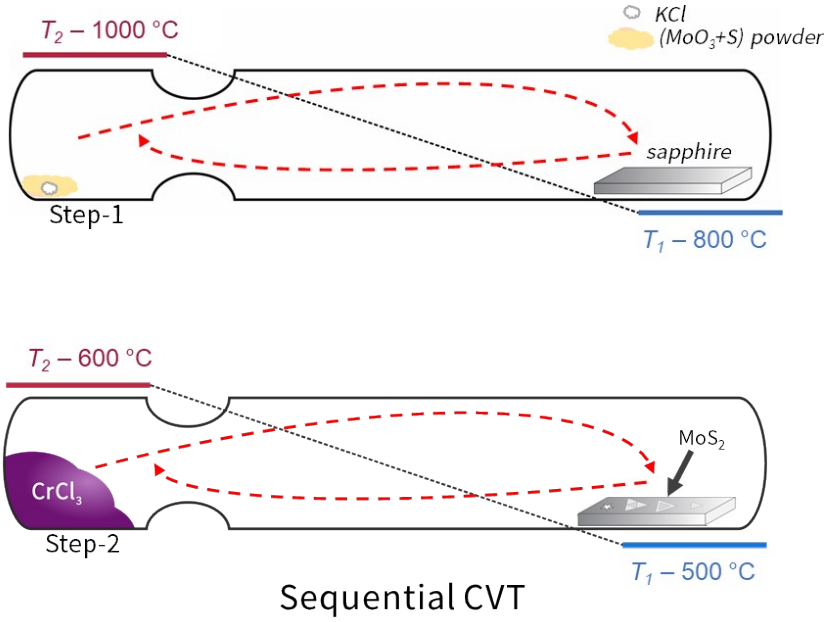

Figure S2. Schematic sequential CVT setup for growth $CrCl_3/MoS_2$ heterostructures (2-chamber fused silica ampoules (L = 120 mm, dA = 6 mm)).

Figure S3a-c, showing scanning electron microscopy (SEM) images of MoS_2, reveals distinct twisted triangular morphologies with varying angular twists. This characteristic formation indicates the precise layer alignment and the high-quality synthesis of the MoS_2 crystals. The sharp and well-defined edges of the triangles further emphasize the uniformity and crystalline nature of the synthesized MoS_2. Some images are shown at a lower resolution due to the charging effect from the sapphire substrate. Additionally, a schematic representation (Figure S3d) illustrates the different morphologies and sizes of the prepared MoS_2 crystals, providing a comprehensive overview of their structural variations.

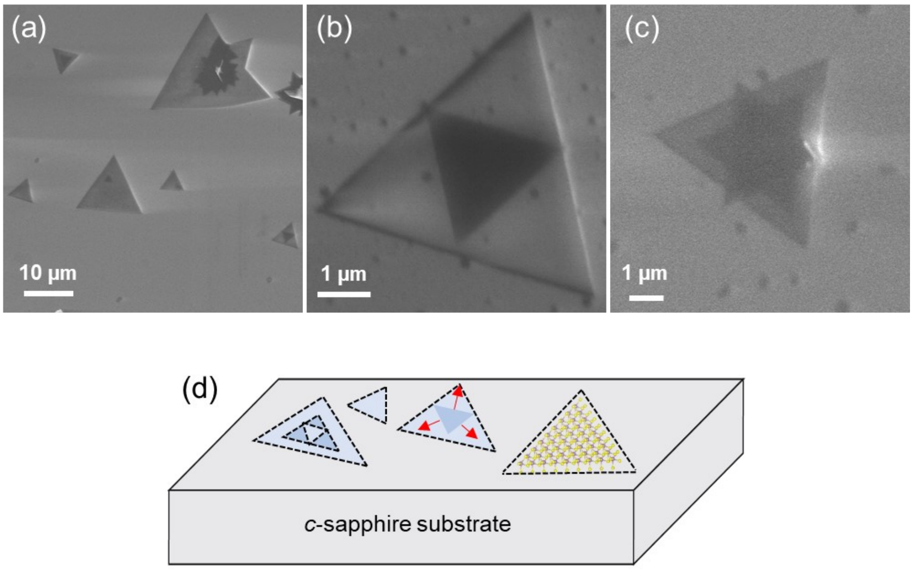

Figure S3. (a-c) SEM images of MoS_2 nanocrystals on *c*-sapphire (0001) substrate. (d) Schematic representation of different morphologies and sizes of the prepared MoS_2 crystals.

Triangular-shaped MoS_2 microcrystals also had visible spots with distorted shapes, where inner triangles touch the outer edges. To analyze if these fragments feature enhanced local strain, we performed Raman mapping for one of such crystals. Figure S4 shows microphotography of the mapped region (a) as well as the maps based on the intensity of the E_{2g} mode between 370 and 390 cm^{-1} and the frequency difference (ΔE) between E_{2g} and A_{1g} modes at 382 and 407 cm^{-1}. Intensity maps roughly corresponds to the observed increase of the thickness in the central part, whereas ΔE is still sensitive to the change of the number of layers (although the crystal is well beyond the monolayer range, where Raman spectra are especially sensitive to the number of layers). Series of the spectra along the lines of constant x or y are shown in Fig. S4 (d-f), and in Fig. S4 (g-i) with higher magnification of the A_{1g} mode. Spectral series along the line x = 12 μm reflects the outward-curved shape of the triangle apex region: whereas only the substrate signal is seen for y ≤ 4 μm and y ≥ 12 μm, small MoS_2 signals are visible for y =6–10 μm. Analogous measurements along the line of x = 10.3 μm already show intense MoS_2 features in the whole series, accompanied by a visible variation of the A_{1g} mode frequency in a thicker region with y = 6–8 μm (see enhancement in Fig. S4i). Series along the line y = 8 μm compares the spectra in the center of the thicker region (x < 9 μm) and at the edge (x = 10.3 μm). The spectra are nearly identical, but the frequency variation of 0.3 cm^{-1} can be seen for the A_{1g} mode at x = 10.3 μm (see enlarged spectra in Fig. S4g, h). The E_{2g} mode is sensitive to strain, and the A_{1g} mode is sensitive to both the electrostatic interactions and exhibits minimal sensitivity to strain [4, 5]. Hence, the shift in A_{1g} modes, which is very small and can originate from the substrate. Consequently, this indicates the absence of significant strain within the MoS_2 crystals.

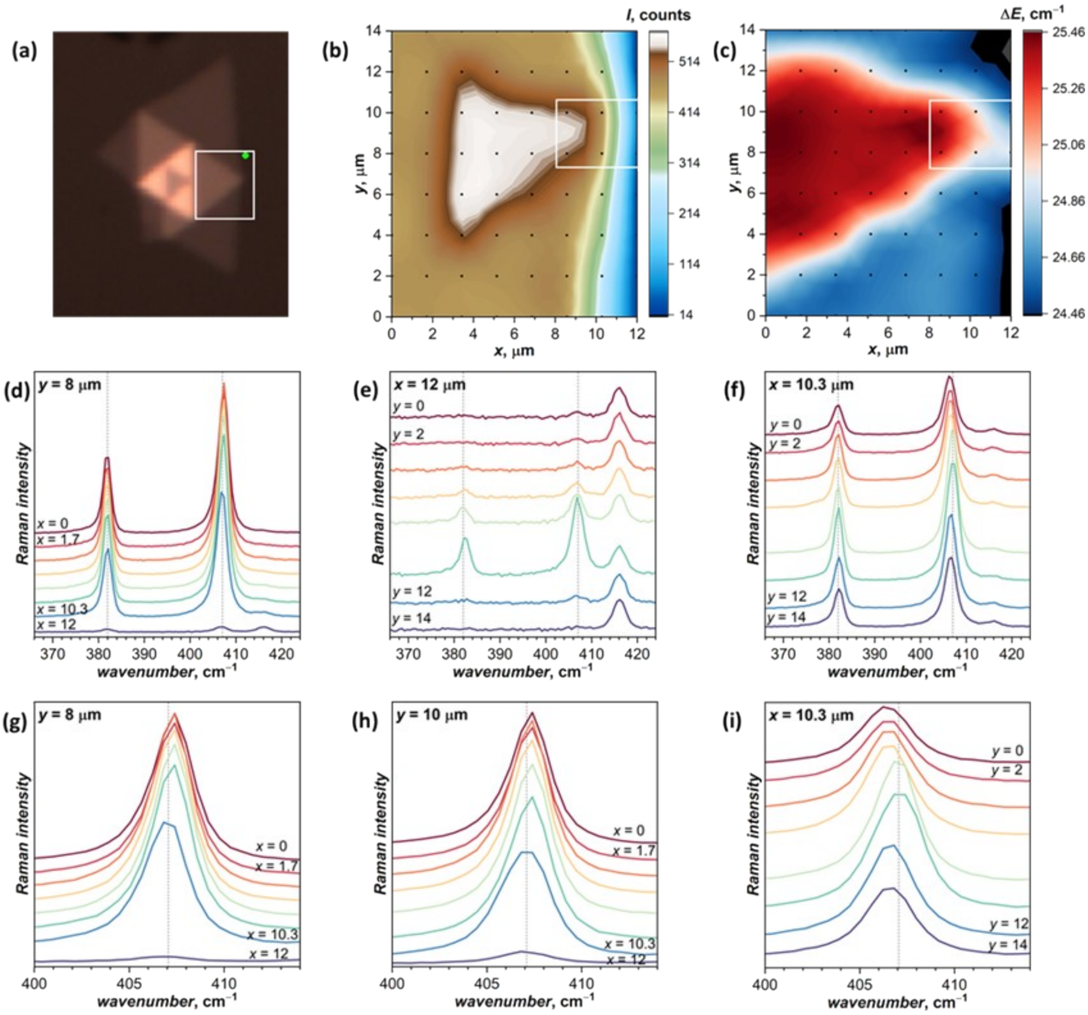

Figure S4. (a) Microphotography of the MoS_2 microcrystal, showing he region, in which Raman mapping was performed. (b) Raman map of the integral intensity of the E_{2g} mode. (c) Raman map of the frequency difference between E_{2g} and A_{1g} peaks. In (c) and (d), black dots are central positions, at which the spectra were measured, while white rectangle shows the region of particular interest as discussed in the text. (d)-(i) Individual spectra along certain x and y directions. Measurements are performed with 532 nm excitation; the size of the laser spot is ca 2 μm.

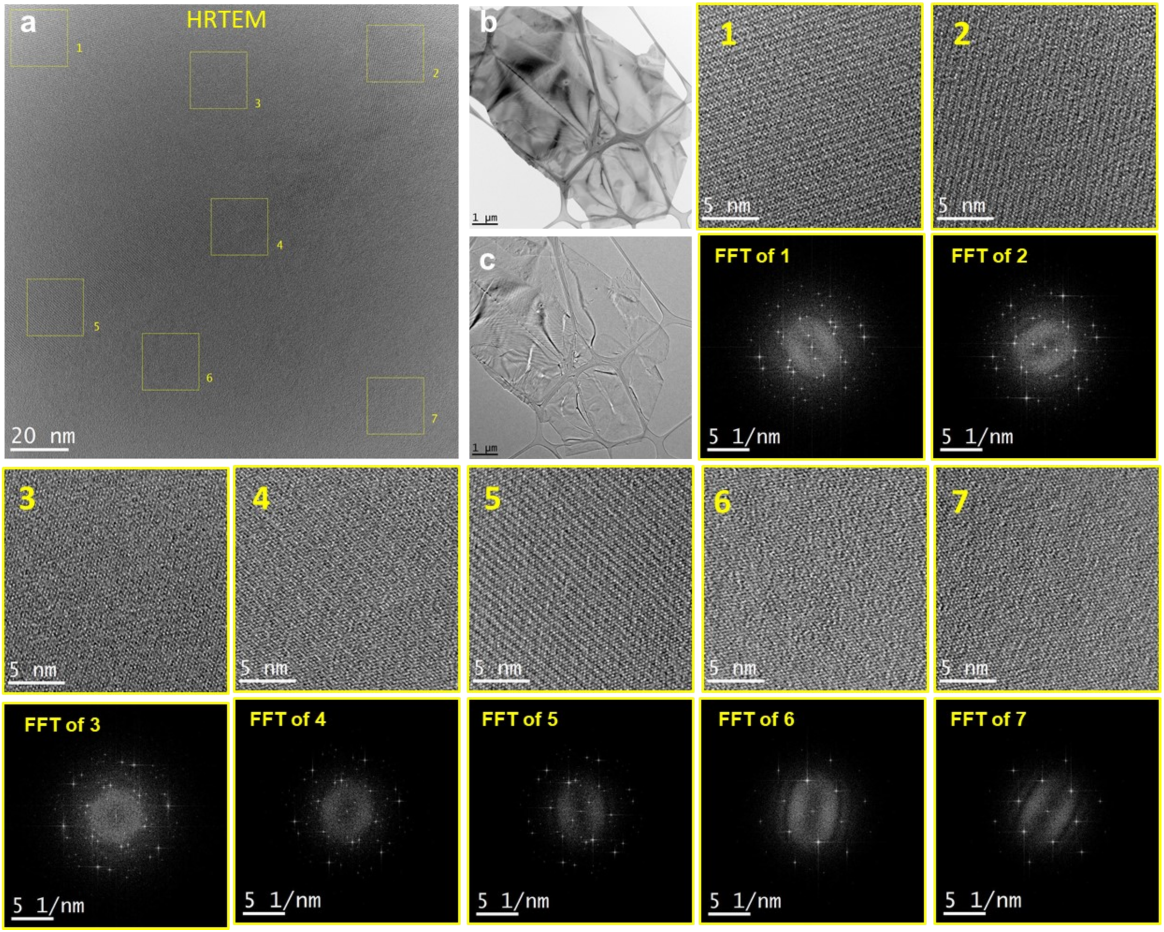

Figure S5. HRTEM of a twisted MoS_2 flake using local fast Fourier transforms (FFTs). (a) HRTEM image with seven different areas (yellow boxes) labeled from 1 to 7. (b,c) Bright-field TEM images with (b) and without contrast aperture (c) from which the HRTEM image (a) is taken. The position of the HRTEM image (a) is indicated as a red square in (b) and (c). (1-7) Sub-images of (a) and their FFTs. The images (1-5) show a superstructure visible as Moiré patterns and as satellite reflections in the corresponding FFTs due to the stacking of two twisted layers. The images (6) and (7) and corresponding FFTs show regions of the flake, where there is only one layer left at the lower edge.

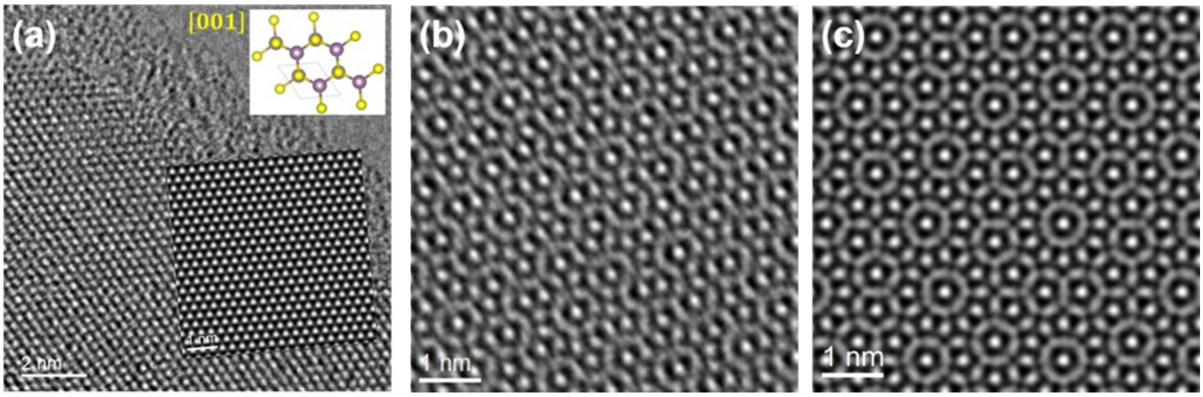

Figure S6. TEM investigations of MoS_2 nanoflakes. (a) HRTEM image of a monolayer region of MoS_2 oriented along [001] zone axis with corresponding atom model and image simulation (insets). (b) HRTEM image of a bilayer MoS_2 region twisted by 26°. (c) Simulated HRTEM image of bilayer MoS_2 twisted by 26°, generated using the DrProbe software [6].

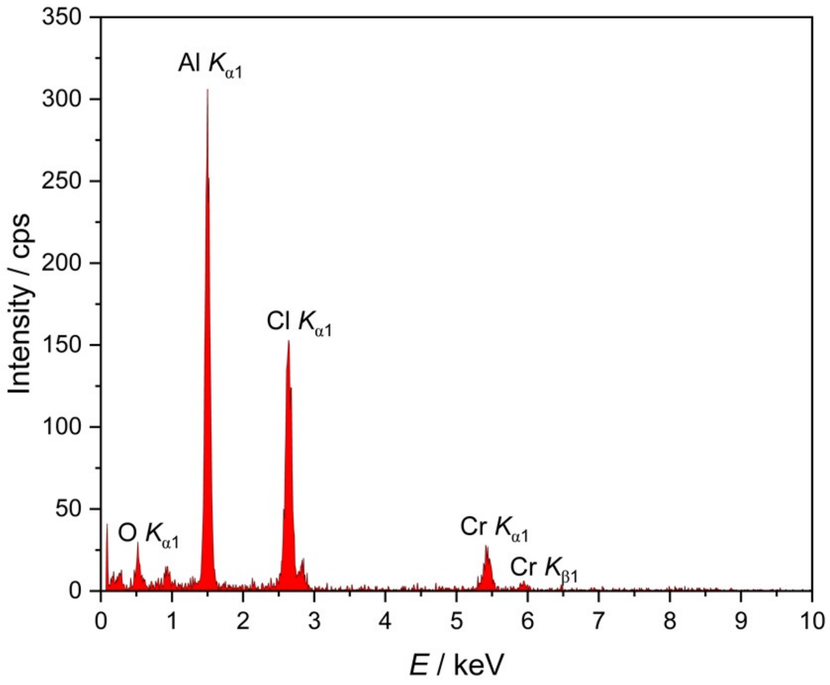

Figure S7. EDX spectrum of a $CrCl_3$ flake. The highest peaks are associated with Al and O, which come from the Al_2O_3 substrate.

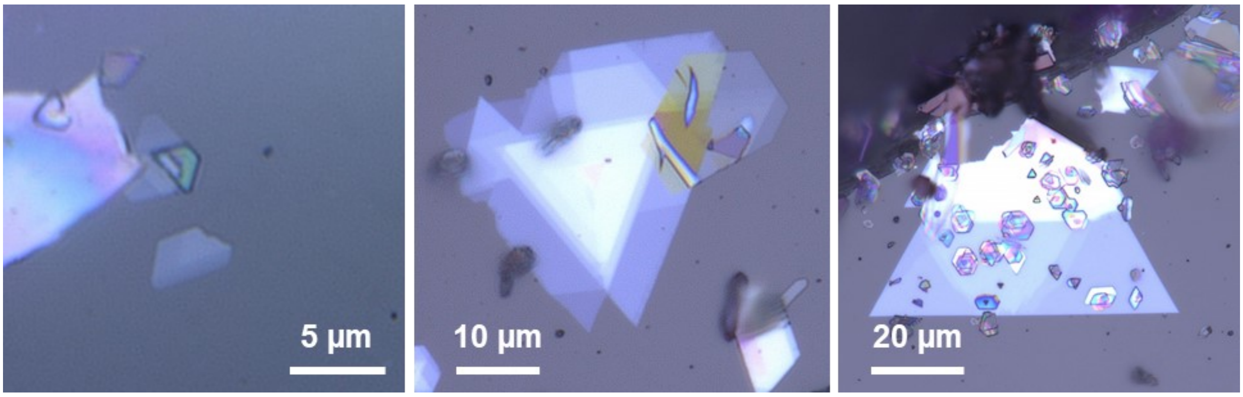

Figure S8. Optical microscope image of $CrCl_3/MoS_2$ prepared by sequential CVT method (MoS_2: trigonal shape, $CrCl_3$: half hexagonal and hexagonal shapes).

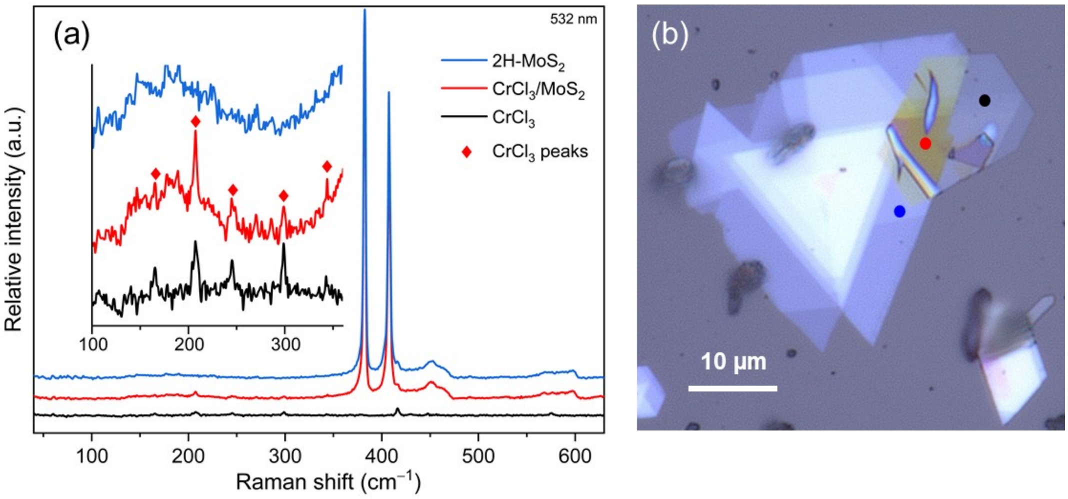

Figure S9. Raman spectra taken from the three different regions (labelled on the optical microscope image b), showing the multilayer triangles of MoS_2, and hexagonal $CrCl_3$ on the top. There are overlapping Raman signature with MoS_2 (red line).

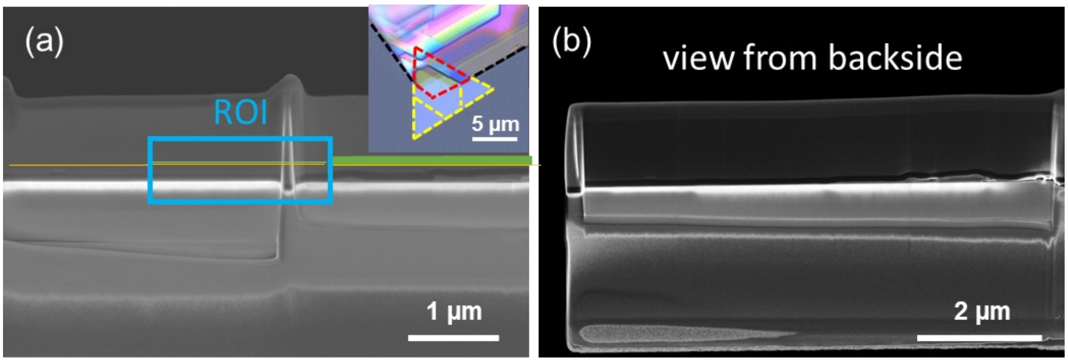

Figure S10. Cross-sectional SEM image of the $CrCl_3/MoS_2$ heterostructure from frontside (a) and backside (b) views after applied focused ion beam (FIB), showing the area selected for cross-sectional TEM analysis. The corresponding optical microscope image (Inset in (a)).

Description of the used CVT simulation model

Typically, the simulation of regular CVT processes is realized by first calculating the thermodynamic equilibria for the source part of the system with the respective set of parameters and consecutively "transferring" only the vapor phase to the sink system, performing there an additional equilibria calculation with the adjusted set of parameters. From the differences of the results in these independent source and sink equilibria, the transport process can be modeled. However, in this routine, which is implemented in the most used CVT simulation software TRAGMIN and CVTRANS, preexisting material in the sink is not thought of, but is crucial for a correct modeling of the sequential heterostructure deposition process. To adapt for this issue, we extended the software code of TRAGMIN to enable adding additional material, in this case a specific amount of MoS_2, to the sink before the equilibrium calculation. If this is done, this means that the first calculation in the source is not incorporating the material that is added in the sink. Still, the use of the additional material in the sink is inevitably also influencing the equilibria in the source (except for the case that the additional material is identical to the material that is going to be deposited in the process anyhow or if it is chemically inert). To further account for this additional influence, additional calculation cycles were added by transferring the equilibrium vapor phase of the sink calculation back again to the initial source system, there again using the "left behind" condensed phases from the first source calculation. This transfer of the equilibrium vapor phase between source and sink is performed until a convergence of the calculated vapor phase amounts for the source and the sink side between two consecutive cycles is reached (convergence criterion is a difference below 1% or an absolute difference small than 10^{-13} mol). This vapor phase transfer process is similar to the

routine for non−stationary transport that is implemented in the software CVTRANS, but extends this concept for the addition of extra sink material [7].

For the case of the simulation of the transport of $CrCl_3$ on top of MoS_2, this results in the following calculation steps:

1. Equilibrium calculation of $CrCl_3$ + H_2O traces in the source

2. Transfer of the source equilibrium vapor phase to the sink

3. Equilibrium calculation of the source vapor phase + MoS_2 in the sink

4. Transfer of the sink equilibrium vapor phase back to the source

5. Repeating the transfer cycle until convergence in source + sink between cycles is reached

When the converged state is reached, the calculated results correspond to a regular stationary transport process. As the number of cycle iterations to reach the convergence is quite small (for the cases described in this publication all below 10), it is likely that the state of stationary transport should be reached shortly after the start of the experiment, potentially even during heating up the system.

The potential formation of a $CrCl_3$−$MoCl_3$ solid solution (assuming ideal solid solution formation) was exemplary considered for several simulations, but the used parameters always lead to the deposition of practically pure $CrCl_3$, so that their potential formation was not further discussed.

The amounts of elements/compounds that were used in the simulations were selected to mimic the actual amounts during practical transport experiments, for example using only $6\cdot10^{-5}$ mmol MoS_2 in the sink to describe only a very thin layer of it on the substrate as the first heterostructure layer.

The following species were used in the simulations:

vapor species: Ar, Cl, Cl_2, HCl, H_2O, H, H_2, O, O_2, S, S_2, S_3, S_4, S_5, S_6, S_7, S_8, S_2O, SO, SO_2, SO_3, H_2S, H_2S_2, SCl, S_2Cl_2, SCl_2, $SOCl_2$, SO_2Cl_2, S_2Cl, $CrCl_2$, $CrCl_3$, $CrCl_4$, CrOCl, $CrOCl_2$, $CrOCl_3$, $CrOCl_4$, CrO_2Cl_2, CrS, $MoCl_4$, $MoCl_5$, $MoOCl_2$, $MoOCl_3$

condensed phases: Cr, $CrCl_2$, $CrCl_3$, Cr_2O_3, CrS (α+β), Cr_2S_3, Cr_3S_4, Cr_5S_6 Cr_6S_7 (α+β+σ), Mo, $MoCl_2$, $MoCl_3$, $MoCl_4$, $MoCl_5$ (s+l), $MoOCl_2$, MoO_2Cl_2, $MoOCl_3$, MoO_2, MoO_3 (s+l), MoS_2, Mo_2S_3

Additional known species for the given element combinations were also considered, but not used due to known irrelevance.

REFERENCES

1. S. Froeschke, D. Wolf, M. Hantusch, L. Giebeler, M. Wels, N. Gräßler, B. Büchner, P. Schmidt and S. Hampel, *Nanoscale*, 2022, **14**, 10483-10492.
2. E. Kahn, Ph.D. Ph.D. Dissertation, The Pennsylvania State University The Graduate School 2020.
3. M. Yoshimoto, T. Maeda, T. Ohnishi, H. Koinuma, O. Ishiyama, M. Shinohara, M. Kubo, R. Miura and A. Miyamoto, *Applied Physics Letters*, 1995, **67**, 2615-2617.
4. J.-U. Lee, S. Woo, J. Park, H. C. Park, Y.-W. Son and H. Cheong, *Nature communications*, 2017, **8**, 1370.
5. G. Haider, M. Gastaldo, B. Karim, J. Plsek, V. Varade, O. Volochanskyi, J. Vejpravová and M. Kalbac, *ACS Applied Electronic Materials*, 2024, **6**, 2301-2308.
6. J. Barthel, *Ultramicroscopy*, 2018, **193**, 1-11.
7. R. Gruehn and R. Glaum, *Angewandte Chemie International Edition*, 2000, **39**, 692-716.
